# rTMS Modulation of Behavioral and Biological Measures in 3xTg-AD Mice

**DOI:** 10.3390/brainsci14121186

**Published:** 2024-11-26

**Authors:** Eric P. Kraybill, Fatemeh S. Mojabi, Alesha M. Heath, Cierra R. Spikes, Charlotte Beard, M. Windy McNerney

**Affiliations:** 1 Mental Illness Research Education and Clinical Center (MIRECC), Department of Veteran Affairs, 3801 Miranda Ave, Palo Alto, CA 94304, USA; 2 Department of Psychiatry and Behavioral Sciences, Stanford University School of Medicine, 401 Quarry Rd, Stanford, CA 94305, USA; 3 Department of Psychology, Stanford University, 450 Jane Stanford Way, Stanford, CA 94305, USA

**Keywords:** Alzheimer’s disease, rTMS, ANA12, disinhibition-like behavior

## Abstract

Background/Objectives: The biological basis for behavioral manifestations of Alzheimer’s disease remains unclear. Emotional and behavioral alterations of Alzheimer’s disease can result in substantial caregiver burden and lack effective management. This study expands upon previous work investigating behavioral alterations in mice with Alzheimer’s disease and a potential treatment of increasing brain-derived neurotrophic factor (BDNF) using repetitive transcranial magnetic stimulation (rTMS). Methods: A total of 47 3xTg-AD (Alzheimer’s) and 53 B6 (wildtype) mice were administered ANA12 (an antagonist of TrkB receptor) or Vehicle (saline) and then rTMS or Sham treatment daily. After 14 days of treatments and injections, mouse behavior was assessed under various behavioral cognitive tests. Mice were then perfused, and brain samples were processed for histology and protein assays. Brain homogenates were analyzed for BDNF and its downstream signaling molecules. Results: Open field testing demonstrated that 3xTg-AD mice spent more time in the center than B6 mice. 3xTg-AD-Sham mice injected with ANA12 were the only group to travel significantly less distance than B6-ANA12-Sham or B6-Vehicle-Sham mice (*p* < 0.05), while 3xTg-AD-rTMS mice (irrespective of injection) were not significantly different from B6 mice. 3xTg-AD mice had significantly greater measured levels of BDNF and TrkB than the wild-type mice. Conclusions: Treatment of Alzheimer’s disease using rTMS positively affects elements of hypoactivity, but not all behavioral abnormalities. rTMS shifted 3xTg-AD open field behavioral test measures, generating significant differences between untreated 3xTg-AD and B6 genotypes. Despite its benefit, further investigation of rTMS as a treatment for Alzheimer’s disease as well as its biological underpinnings are needed.

## 1. Introduction

Approximately one in nine individuals 65 years and older in the United States suffer from Alzheimer’s Disease (AD), with an estimated societal cost of around USD 345 billion in 2023 [1]. AD causes irreparable damage to the brain, leading to significant cognitive decline and eventual death [2]. Current cognitive tests for AD and mild cognitive impairment (MCI), including the Alzheimer’s Disease Assessment Scale-cognitive (ADAS-Cog), focus primarily on memory [3,4], but AD encompasses a variety of cognitive deficits in addition to memory loss. Other behavioral changes must additionally be considered in order to recognize and provide effective support for those suffering from the disease [5]. Emotional and behavioral changes can result in substantial caregiver burden, and lack effective management [6,7].

Repetitive transcranial magnetic stimulation (rTMS), a form of treatment that utilizes a magnetic field to stimulate cortical regions of the brain, has been shown to support neural plasticity, indicating its potential utility to help address a variety of neurobiological conditions including AD [8]. Studies demonstrating the effectiveness of rTMS in improving the neurobiological functioning of patients with treatment resistant depression have led to its adoption in clinical care [9]. Similarly promising results for MCI and cognitive decline [10] make rTMS an attractive modality to study for AD.

Some research has evaluated the effects of rTMS on regions implicated in emotional regulation and behavioral control. For example, a study by Berlim et al. (2013) [11] demonstrated that high-frequency rTMS (HF-rTMS) and electroconvulsive therapy (ECT) can reduce depressive symptoms. These symptoms often share some neurocircuitry features with apathy-like behaviors, such as poor emotional regulation, observed in other disorders like borderline personality disorder (BPD) [12]. Recent studies have explored the effects of rTMS on brain regions involved in emotional regulation [13], highlighting its potential impact on emotional behavior.

rTMS is thought to increase the release of brain-derived neurotrophic factor (BDNF), a key protein related to various cognitive behaviors by supporting neural communication, growth, and survival [14,15,16,17]. Therefore, if BDNF is critical to the biochemical cascades affected by rTMS then blocking the receptor for BDNF, TrkB, may reveal the mechanism by which rTMS supports cognition. Alternatively, some researchers argue that abnormal increases in neural activity is associated with more impulsive behavior [18]. While rTMS has been shown to improve cognition and increase BDNF, its relationship with BDNF and behavioral control in AD has yet to be established.

This study aims to expand on recent reports of abnormal behavior in 3xTg-AD mice including reduced velocity but increased time in the center of an open field task which have been linked to apathy or disinhibition [19,20,21]. Using characterization of abnormal behavior in the open field as reported previously [19], the effects of rTMS and BDNF on behavioral changes in an AD mouse model were explored. Twelve-month-old 3xTg-AD (3×) or B6129SF2/J wild type (B6) mice were injected with a non-competitive TrkB receptor antagonist, ANA12 [22], or Vehicle (saline/control) 2 h before treatment with rTMS. By inhibiting the BDNF pathway, ANA12 could be a key agent in revealing the mechanism through which rTMS influences behavior. We hypothesized that rTMS would reduce hypoactivity and time spent in center in the open field task, while ANA12 would abolish this effect.

## 2. Materials and Methods

### 2.1. Animals

This study included 100 female mice (53 B6 and 47 3×) aged 12 months. We chose to focus only on female mice because male mice show inconsistent pathology with minimal plaque and tangle deposition even by 12 months of age [23]. While there is some concern that estrus phase may influence behavior, a recent publication showed that behavior is more variable in male mice, and there was no influence of estrus cycle in younger female mice [24]. However, we determined estrus phase in our mice as an extra precaution. Mice were maintained on a 12 h light/dark cycle with ad libitum access to food and water in a temperature and humidity-controlled environment. All procedures were approved under the institutional and animal care and use committee (IACUC) at the Department of Veterans Affairs, Palo Alto, California. Research ethics policies specifically surrounding rodents are outlined within the Department of Veterans Affairs [25] and can be found at the following website: https://www.va.gov/ORO/rsaw.asp, accessed on 15 November 2024.

### 2.2. ANA12 and rTMS Treatment

Our rTMS coil stemmed from methods pioneered in the Roger Laboratory [26,27] and optimized for our research [28] and consists of a copper wire solenoid with an outer diameter of 8 mm and an inner diameter of 6 mm. To deliver stimulation without the need for restraint or anesthesia, a coil support was attached to the mouse skull so the coil could be clipped in place. A 1–2 cm incision was made on the scalp, and the support was attached over bregma using LockTite glue and dental cement, followed by suturing the wound closed. After a recovery period of 5 days, mice were habituated to the stimulation arena on day 1 and then the coil on days 2 and 3 (10 min each day). Stimulation was consistent with previously published methods [16,17], running at 10 Hz for 10 min for a total of 6000 pulses per day over 14 days (intensity~12 mT). Magnetic field strength around the coil was previously measured and modeled, showing a peak strength of 21 mT and a calculated peak electric field of 5 V/m [28]. With identification of the mouse dorsolateral prefrontal cortex (DLPFC) presenting a challenge, the coil was designed to target the frontal cortex, with a 50% field intensity reduction occurring at 24 mm, covering approximately half of the area of the cortex. Previous studies have consistently demonstrated the designed coil’s ability to produce biochemical changes in the body [16,17,28]. Sham mice were treated the same as the rTMS groups with a coil attached to the coil support, but no stimulation was delivered. ANA12 (Sigma, St Louis, MO, USA) in 1% DMSO and sterile PBS or Vehicle control (1% DMSO in PBS) was injected intraperitoneally at 0.5 mg/kg 2 h prior to treatment [22]. Sample sizes per group are shown in Table 1 below.

### 2.3. Behavioral Tasks

The day after the completion of the 14-day rTMS treatment, mice underwent a series of cognitive-behavioral tests over 4 days, all starting at 09:00 am. Mice completed the Open Field Task (OFT) on day one, followed by the Object Recognition Test (ORT) on day two, the Object Place Task (OPT) on day three and the Y-maze task on day four following a similar timeline to McNerney et al. (2023) [17]. For the OFT task, mice were placed in a 50 × 50 cm arena for 10 min. This also served as the habituation for the ORT and OPT. The recognition tasks consisted of a 10 min object familiarization phase, followed by a 3 h wait time, and then 10 min for novel object exploration. The Y-maze task consisted of placing the mice in the center of the Y-maze and allowing them to explore for 5 min. Activity in the OFT was quantified using automated TopScan software (version 3.0) and object exploration time and entrances into Y-Maze arms by experimenters blinded to condition.

24 h following the completion of all behavioral tasks, vaginal smears were taken to assess estrus cycle before mice were transcardially perfused with cold PBS. After perfusion the brain was extracted, and the right hemispheric hippocampus and cortex were isolated and stored in minus 80 °C until homogenization. The left hemisphere of the brain was kept in 4% paraformaldehyde solution (PFA) at 4 °C for 48 h and then transferred to 30% sucrose in PBS at least 48 h prior to cryosectioning into 30 μm sections for immunofluorescent (IF) staining.

### 2.4. Biochemical Measures

All samples were run in duplicate, and any samples with high variability between duplicates or outside the range of detection were rerun. Hippocampi were homogenized in 400 μL cold Syn-Per synaptic Protein Extraction Reagent (Thermo Scientific, Waltham, MA, USA) containing 1× Protease and Phosphatase inhibitor tablets (Roche, Indianapolis, IN, USA). The synaptic (TrkB) and cytosolic (AKT, ERK, PLCγ) hippocampi fractions were used for Western blot for TrkB and downstream proteins (AKT, ERK, PLCγ) following protocols detailed in McNerney et al. (2023) [17]. Briefly, samples were diluted in tris-buffered saline and denatured by boiling at 95 °C for 5 min with 1:40 mercaptoethanol. Electrophoresis was run on precast gels (BioRad, Hercules, CA, USA) at 100 V and proteins transferred via the Transblot Turbo System (BioRad) prior to blocking (Rockland, Limerick, PA, USA). Membranes were incubated overnight with the following antibodies: TrkB (1:500, BD, Franklin Lakes, NJ, USA), ERK1/2 (1:1000, Santa Cruz, Dallas, TX, USA), AKT (1:500, Cell Signaling, Danvers, MA, USA), and PLCγ (1:500, R&D, Minneapolis, MN, USA). Cofilin (1:2000, Sigma, St Louis, MO, USA), and β-actin (1:1000, ThermoFisher) were used for the standard loading controls. Blots were incubated in secondary antibodies (Licor, Lincoln, NE, USA) for 1 hr at RT and imaged (Odyssey Image Study version 5.2, Lincoln, NE, USA). Bands were quantified using Gel Analyzer (GelAnalyzer, version 19.1). TrkB, ERK, AKT, and PLCγ were normalized to their respective loading.

The full fractions of the hippocampus homogenate were diluted 1:10 in sample dilution buffer and measured in an enzyme-linked immunosorbent assay (ELISA) for BDNF analysis (R&D) in accordance with the manufacturer’s instructions and in a similar manner to previous publications [16]. Raw absorption (OD450) data from ELISA were analyzed through construction of a four-parameter logistic curve (myassays.com, accessed on 6 October 2023). BDNF was normalized to bicinchoninic acid (BCA; Pierce BCA Protein Assay Kit, ThermoFisher, Waltham, MA, USA).

### 2.5. Immunofluorescence Staining

In accordance with McNerney et al. (2022) [16], to confirm the presence of plaques and tangles in the 3× mice, a dual staining procedure for Aβ and phosphorylated-tau was conducted with Aβ (dilution 1:1200, Cell Signaling, Danvers, MA, USA) and AT8 (dilution 1:1200, ThermoFisher, Waltham, MA, USA) antibodies. Floating brain slices (30 μM thick) were first rinsed in 1× PBS, followed by permeabilization in PBS containing 0.3% triton and then blocking in 10% normal goat serum. The slices were then incubated with the primary antibodies overnight at 4 °C, rinsed in PBS, and subsequently exposed to secondary antibodies 1:500 (ThermoFisher, Waltham, MA, USA) for 1 h at room temperature. DAPI 1:1000 was introduced during the final rinse. Slices were mounted and cover slipped using Fluoromount (Invitrogen, Waltham, MA, USA) for imaging under a Keyence microscope. Quantification of plaques and tangles was performed using ImageJ software (version 2.14.0/1.54f) in the dorsal cortex, ventral cortex, septal nucleus, hippocampus, and subiculum and normalized to the total area of each region examined.

### 2.6. Statistics and Analysis

Behavioral and biochemical measures were tested and met the normality assumption (skewness, kurtosis and Shapiro–Wilk of the calculated residuals). Data were examined using a three-way ANOVA, looking at the effect of genotype (3× or B6), treatment (rTMS or Sham) and injection (ANA12 or Vehicle). Post hoc pairwise comparisons were performed between all groups and adjusted using Tukey (significance at *p* < 0.05). If the homogeneity of variance was violated, significance between groups was instead determined with the Welch Test and subsequently post hoc corrected with Games–Howell; these cases are noted in the results. The data for immunohistochemical staining were non-parametric, and therefore, differences between groups were tested with Kruskal–Wallis test. All statistics were performed using SPSS (version 22, IBM, Armonk, NY, USA) and graphs produced using GraphPad (version 7, Prism, La Jolla, CA, USA).

## 3. Results

### 3.1. Model Characterization

Table 2 shows the percent mice in each estrus phase. Estrus cycle was not associated with condition (*p* > 0.05) nor related to any of the measures of interest (all *p* values > 0.05); see Supplementary Table S1 for a correlation table. Therefore, no mice were excluded based on estrus phase and phase information this was not included in the analyses, similar to McNerney et al., 2022 [16].

**Figure 1:**
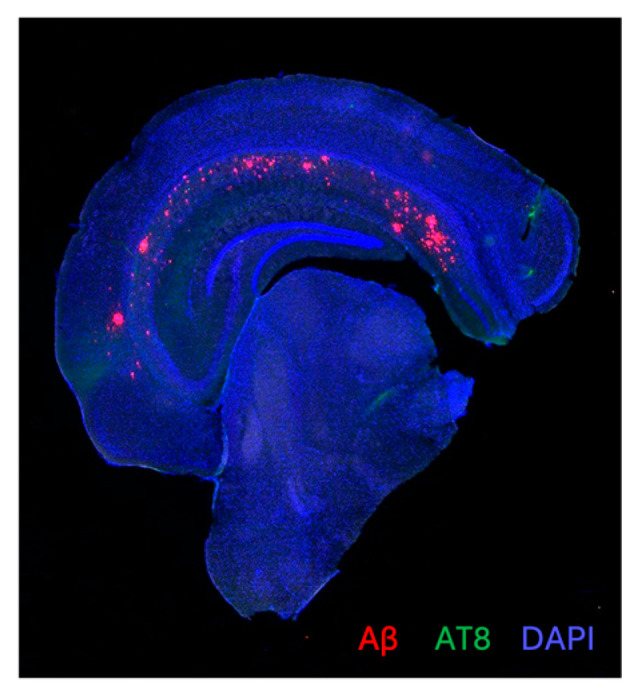
Representative stain of AD pathology in 3× mouse.

The 3× mice were noted to have plaques and tangles, consistent with the AD phenotype and shown in Figure 1. The greatest plaque load was found in the septal nucleus and subiculum. There was no difference in the plaque load in the dorsal cortex, ventral cortex, septal nucleus, hippocampus, and subiculum between the different experimental groups (Kruskal–Wallis, *p* > 0.05; Table 3). Therefore, ANA12 and rTMS had no influence on plaque load.

### 3.2. Behavior

**Figure 2:**
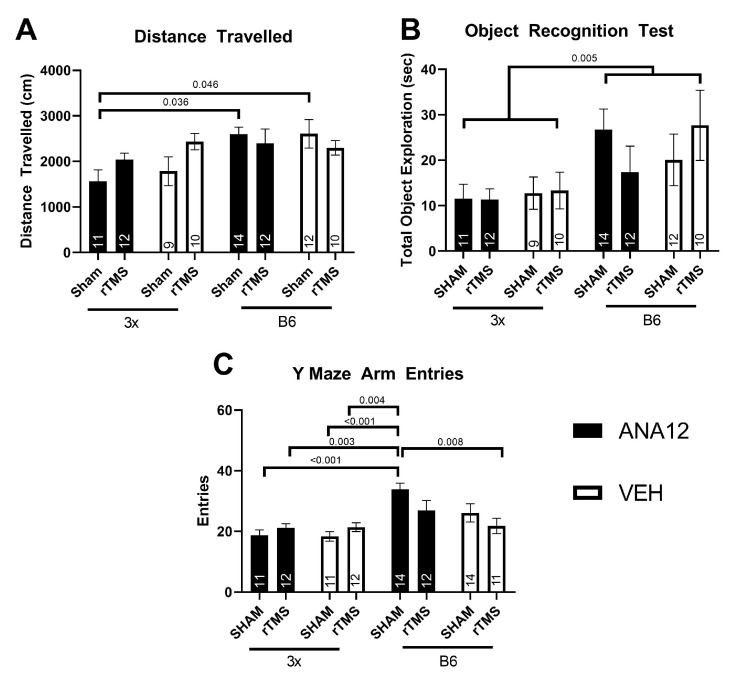
Lower exploratory activity in 3× mice was shown in the distance travelled in the Open Field Test (**A**), in the total object exploration in the familiarization phase of the Object Recognition Test (**B**) and in the total entries into arms in the Y Maze (**C**). Annotations in bars are the n in each group and the Tukey or genotype effect *p* values of significant results are shown.

**Figure 3:**
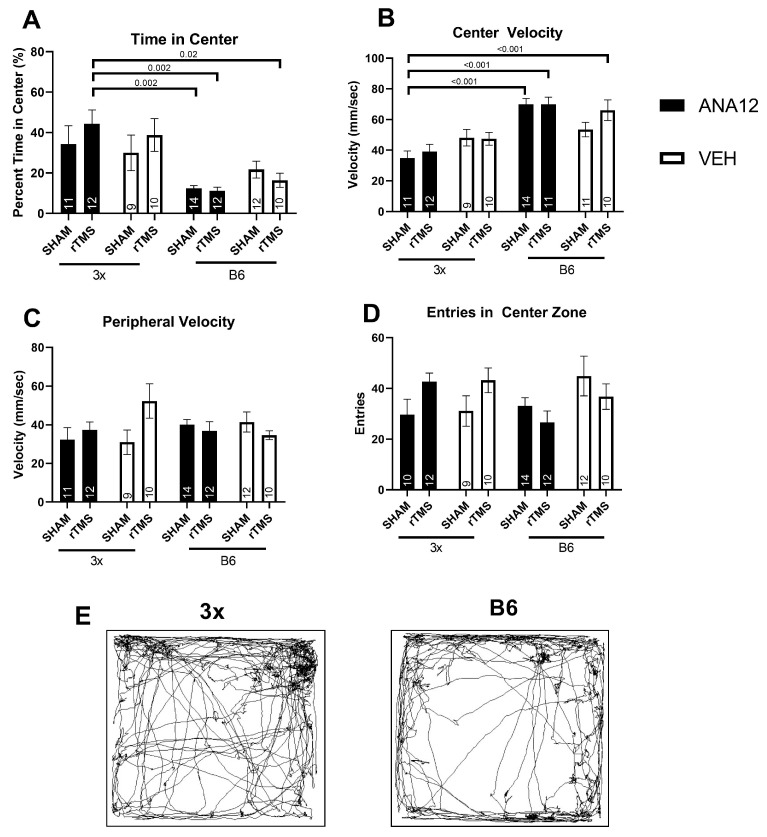
Behavioral disinhibition phenotype was present in the 3× mice. In the center 3× spent more time (**A**) and had a slower velocity (**B**) than B6 mice; however, these differences were not present in the periphery (**C**) for the Open Field Test. Number of entries into the center zone between conditions (**D**). Represented trace images of activity from one 3× and one B6 mouse are shown (**E**). Annotations in bars are the n in each group and the Tukey *p* values of significant results are shown.

Two mice (1 3× and 1 B6) were excluded from behavioral analysis for consistent inactivity throughout all tasks. Due to an equipment malfunction, behavior from eight mice was not analyzable (4 3× and 4 B6) in the OFT. For the analysis of center velocity two mice (1 3×, 1 B6) were excluded for being extreme outliers (>3SD’s from the mean), leaving 90 mice for all OFT tasks measurements, except center velocity (88 mice). The number of mice per condition analyzed in the behavioral tasks are shown in Figure 2 and Figure 3.

First, the total distance traveled in the open field test over the ten-minute testing period was investigated to determine overall exploration differences between groups. An interaction effect between genotype and treatment was found on the total distance (three-way ANOVA effect of genotype × treatment *F*(1,82) = 5.81 *p* = 0.018, partial η^2^ = 0.066), the genotype effect is consistent with McNerney et al., 2023 [17] (Figure 2A). Post hoc testing revealed that 3x-ANA12-Sham mice traveled less distance than B6-ANA12-Sham or B6-Vehicle-Sham mice (Tukey’s *p* < 0.05); however, the 3x-ANA12 mice treated with rTMS had an increase in distance traveled such that they were not statistically different from B6-ANA12-Sham or B6-Vehicle-Sham mice (*p* > 0.05). To further characterize overall activity and exploration, we analyzed the exploration time in the ORT and the total entries in the Y-Maze tasks. For the familiarization phase of the ORT, there was a significant effect of genotype (Three Way ANOVA Effect of Genotype *F*(1,82) = 8.51, *p* = 0.0025 partial η^2^ = 0.094), suggesting that 3× mice explored object less than the B6 regardless of treatment (Figure 2B). In the Y-maze, there was an interaction between genotype and treatment in the number of entries in the Y-maze task (three-way ANOVA effect of genotype × treatment *F*(1,89) = 6.59, *p* = 0.012, partial η^2^ = 0.069, Figure 2C). Post hoc tests revealed B6-ANA12-Sham mice had a greater number of entries than all 3× groups and also more than B6-Veh-rTMS (Tukey’s *p* < 0.05). Lower exploration in AD mice is consistent with previous research [29,30], but given the extent of the exploration differences and the number of mice that did not reach criteria for inclusion in the NOR task, quantification of memory performance was not considered appropriate. Similar results were found in the OPT. We recognize this as a shortcoming to be researched further but have previously demonstrated memory deficits with this model [17].

To determine if the reason for this difference in distance traveled was due to locomotor differences or due to a behavioral phenotype, the difference in activity in the center and periphery of the open field was explored. Overall, there was a significant difference between groups for the time spent in the center (Welch Test *F*(7,82) = 5.75 *p* < 0.001, Figure 3A). Post hoc tests revealed that 3x-ANA12-rTMS mice spent a significantly greater amount of time in the center compared to B6-ANA12-Sham (Games-Howell, *p* < 0.01); B6-ANA12-rTMS (Games–Howell, *p* < 0.01), and B6-Vehicle-rTMS mice (Games-Howell, *p* < 0.05). Furthermore, for velocity in the center zone, there was a significant interaction between genotype and injection (three-way ANOVA effect of genotype × injection *F*(1,80) = 9.31 *p* = 0.003 partial η^2^ = 0.10, Figure 3B). Post hoc testing revealed that 3x-ANA12-Sham mice moved significantly slower in the center zone compared to B6-ANA12-Sham (Tukey *p* < 0.001), B6-ANA12-rTMS mice (Tukey *p* < 0.01), or B6-Vehicle-rTMS mice (*p* < 0.01). In the periphery, no effect of injection was found for velocity (Three Way ANOVA Effect of Injection (*F*(1,82) = 0.50 *p* = 0.48 partial η^2^ = 0.006), but there was a significant interaction between genotype and treatment (Three Way ANOVA Effect of Genotype × Treatment *F*(1,82) = 6.39 *p* = 0.014 partial η^2^ = 0.072, Figure 3C). Post hoc testing revealed no individual differences for velocity in the periphery between groups (Tukey *p* > 0.05). Finally, there was a significant interaction of genotype and treatment on the number of entries into the center (three-way ANOVA effect of genotype × treatment *F*(1,82) = 7.30 *p* = 0.008 partial η^2^ = 0.08, Figure 3D); however, there were no post hoc differences (Tukey *p* > 0.05). These results indicate that the genotype effect on the total distance traveled may be responsible for differences of behavior in the center zone of the open field test, but not the periphery.

### 3.3. Biological

**Figure 4:**
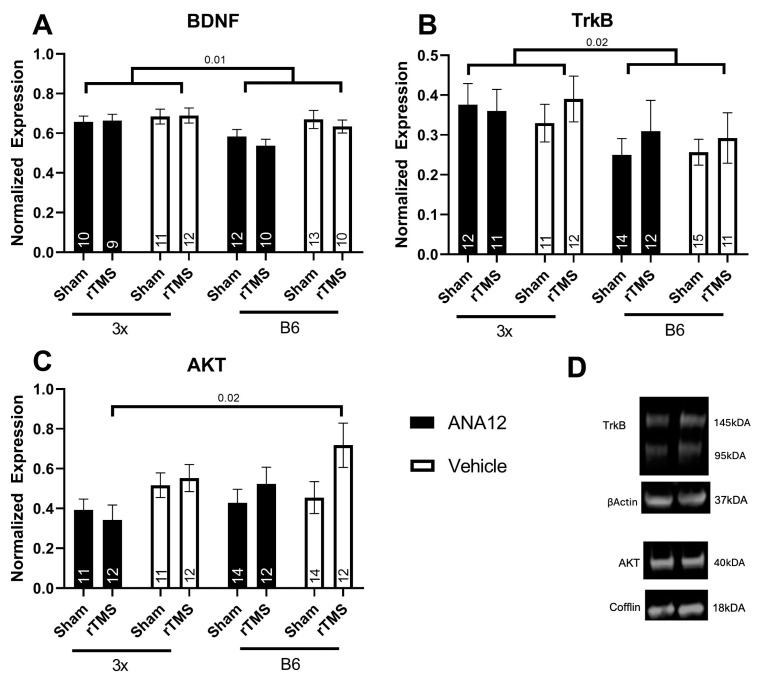
Genotype and ANA12 injections had differing effects on BDNF (**A**), TrkB (**B**) and downstream BDNF-TrkB pathway protein AKT (**C**). Example Western blot bands from TrkB and AKT (**D**). For full unaltered images of Westerns, see Supplementary Figure S1. Annotations in bars are the n in each group and the Tukey or genotype effect *p* values of significant results are shown.

In the hippocampus, the effect of rTMS and ANA12 on BDNF and its subsequent downstream signaling pathways was investigated. For hippocampal BDNF, there was an effect of both genotype (three-way ANOVA effect of genotype *F*(1,87) = 6.57 *p* = 0.012 partial η^2^ = 0.077, Figure 4A) and of injection (three-way ANOVA effect of injection *F*(1,87) = 5.00 *p* = 0.028 partial η^2^ = 0.060), but post hoc testing did not reveal any individual differences between groups (Tukey *p* > 0.05). Western blot data revealed that ANA12 had no influence on TrkB protein expression, but there was a significant effect of genotype (three-way ANOVA effect of genotype: *F*(1,89) = 6.13 *p* = 0.015 partial η^2^ = 0.064, Figure 4B), which replicated McNerney et al. (2023) [19]. There were also no effects of interest for PLCγ and ERK (*p* < 0.05), but there was significant effect of ANA12 injection on AKT (three-way ANOVA effect of injection, *F*(1,90) = 6.31 *p* = 0.014 partial η^2^ = 0.066, Figure 4C), with post hoc tests indicating that 3x-ANA12-rTMS have less AKT than B6-Vehicle-rTMS (Tukey *p* < 0.05). Whilst there were no effects of rTMS, ANA12 injection had a specific impact on AKT and BDNF, indicating that our injections had some influence on TrkB function.

## 4. Discussion

The current study expanded upon previously reported behavioral differences between AD and non-AD mice [19] by utilizing a more advanced stage of AD (12 months vs. 6 months of age), and assessing BDNF-mediated effects of rTMS as a treatment of AD. Results demonstrate that 3× mice exhibit abnormal behaviors, manifested by greater time but decreased velocity in the center for the open field test, consistent with previously established results [19,20,21]. While the overall effect was similar to behavior defined as disinhibition by Hebda et al., 2013 [19], the present study differed in the measured distance traveled, where distance traveled for Sham-3× was significantly less than B6. Although it is unclear why this discrepancy occurred, this could be due to the older age of our mice or the experimental manipulations utilized to increase or decrease BDNF via rTMS or ANA12 injections, respectively. Center avoidance is an innate behavior previously established as a response to anxiety, thought to confer a survival advantage [31,32]. While it has been previously thought that AD mice show less time in the center while engaged in the OFT over a longer period [33], recent studies have also indicated they show a similar behavioral phenotype as measured here [19,20,21]. Not only do 3× mice lack this survival advantage, but also their locomotor function is diminished in the center as reflected by their velocity. However, there velocity was not slower in the periphery, reflecting more of a behavioral over motor deficit. This may be due to increased amyloid in the septal nucleus and subiculum, areas associated with apathy [34]. Similar behavior is seen in humans with advanced AD [35]. Future studies should more comprehensively characterize this behavior in terms of anxiety or other possible explanations.

These behavioral phenotypes in the 3× mice were influenced by rTMS and ANA12 in specific contexts. In 3× mice treated with rTMS, irrespective of injection (ANA12 vs. Vehicle), the total distance traveled was found to be comparable to that of B6 mice under all conditions (Figure 1), indicating an improvement on exploration in these mice. However, when examining the behavioral phenotype, 3× mice injected with ANA12 and treated with rTMS continued to spend more time in the center and had a slower velocity than all B6 mice except B6-Vehicle-Sham, suggesting no further effect on behavioral irregularities in these conditions [22]. For the effect of blocking BDNF signaling with ANA12 on this behavioral phenotype, regardless of treatment (rTMS or Sham), 3× mice injected with ANA12 had significantly reduced velocity in the center compared to B6 mice injected with ANA12, indicating a possible exacerbation. Therefore, it appears that while rTMS may have influenced overall distance traveled, ANA12 reduced the center velocity in the 3× mice, indicating separable effects between rTMS and TrkB antagonism. Exploration of behavioral abnormalities through alternative behavioral testing such as the go/no-go task may provide additional context to the observed changes [36].

In terms of biochemistry, injections of ANA12 did not directly influence TrkB but did result in reduced expression of one downstream component, AKT. While previous studies have uncovered an effect of ANA12 on TrkB phosphorylation [37], the timeframe of this study did not permit this measurement, but given the decrease in AKT, it is likely that manipulations in this study had a similar effect. Our previous research demonstrated that rTMS influenced ERK and PLCγ but not AKT [17], therefore the specificity of the inhibitory effect of ANA12 and antagonizing effect of rTMS should be investigated further to understand the BDNF-mediated mechanism of rTMS. Similarly, BDNF was associated with genotype and injection, such that ANA12 reduced BDNF only in B6 mice. This may be an indication of an alteration in TrkB function in 3× mice, possibly from the increase in the Truncated-TrkB isoform previously reported in this model [17].

A minimal effect of rTMS was found on the OFT and biochemical measurements in B6 mice, consistent with our own publications and that of other researchers [16,17,19]. However, it is intriguing that the only consistent effect of injection on BDNF was in B6 mice. Only the B6-Vehicle-Sham mice were not significantly different than the 3× mice in terms of central velocity, warranting further investigation of downstream pathways and behavioral consequences of blocking TrkB in B6 mice. Previously, rTMS has been shown to influence BDNF in 3× mice [16,17], and there was only a small but insignificant trend for an effect of TrkB in all mice injected with Vehicle. The added manipulations of daily intraperitoneal injections in general may have exerted an influence on the 3× mice, although the behavioral response to daily injection has yet to be systematically investigated on these mice. Therefore, future investigations should consider injecting and providing rTMS less frequently, waiting for a longer period of time between injection and rTMS, or other means of blocking TrkB. Regardless, rTMS did influence the total distance traveled for 3× mice, indicating this effect is related to a biochemical pathway outside of BDNF. Similarly, the influence of ANA12 on center velocity was not directly related to BDNF or TrkB. While there may have been some correspondence with changes in AKT, this cannot be fully established with the current data. However, low AKT may reduce inflammation in AD mice [38] but is also associated with increased anxiety [39]. Therefore, future research should more specifically investigate these pathways with the abnormal behavioral phenotype. Overall, the 3x-ANA12-rTMS groups showed the greatest time in the center, such that blocking TrkB while adding rTMS was associated with a pronounced hypoactive effect. Therefore, disrupting a biochemical component of increased cell firing from rTMS may have been harmful to survival-based behavioral outcomes warranting further investigation.

While it is difficult to map mouse behavioral measurements onto human emotional state, researchers have linked slower velocity and increased time in the center to behavioral disinhibition [19] or apathy [20,21], both of which are characteristic of AD patients [35]. Behavioral disinhibition in AD mice has been defined as a lack of fight of flight behavior when a rodent is placed in a situation where cognitive competence would indicate a protective response [19]. On the other hand, apathy is considered a reduction in goal-directed behavior [20,21], which is consistent with a lack of exploration in our recognition and Y-Maze tasks. rTMS seemed to restore mobility loss in distance traveled, and perhaps an aspect of apathy was also impacted. However, it is difficult to directly tease apart subtle changes in behavior, it alters. While the OFT can be used to characterize abnormal behavior, further research using a specific suit of anxiety and depression-like behaviors should be conducted to further elucidate the nature of the observed phenotype.

Despite the significant results, the various behavioral and biological tests performed included some limitations. Various behaviors could result from factors other than the defined apathy-like or disinhibition behavior, and despite an association, a causal link between neurodegeneration and hypoactivity has yet to be established. Hypoactive behavior may not be solely regulated via the BDNF induced TrkB pathway, which could explain why ANA12 had no effect on the ability of rTMS to reduce time in the center, despite the tendency of rTMS to increase total distance traveled. We also recognize that despite our previous reporting of memory deficits in ORT in these same mouse line, mice from this study did not meet criteria to evaluate memory and do consider this a weakness. We aim to more comprehensively evaluate the circumstances in this data for future publications but believe the behavioral effects found merit dissemination into the scientific community. Finally, the small sample size may have dampened the results, so further confirmatory testing should be performed.

## 5. Conclusions

This study demonstrated a variable response to rTMS in aged 3× mice, indicative of a potential treatment for some of the neurodegenerative behavioral symptoms of AD (e.g., exploration). However, the abnormal behavioral phenotype characterized by apathy or disinhibition-like behavior was not significantly improved by rTMS, irrespective of condition (ANA12 versus Vehicle). Treatment of AD using rTMS positively affects one manifestation of AD even if not all behavioral abnormalities are addressed. Overall, this research has uncovered new questions regarding behavioral abnormalities, disinhibition, or apathy in AD by investigating a possible treatment. The data have resulted in some intriguing findings that merit further investigation including: (1) the specificity of ANA12 on AKT and the differential effect of ANA12 on BDNF in AD mice; (2) the behavioral dissociation between rTMS and TrkB antagonism; and (3) the relationship between BDNF and other factors in hypoactive behavior.

## Figures and Tables

**Table 1:** Samples size per group.

Genotype	Treatment	*n*
3×	ANA12-rTMS	13
	ANA12-Sham	12
	Vehicle-rTMS	11
	Vehicle-Sham	11
B6	ANA12-rTMS	12
	ANA12-Sham	14
	Vehicle-rTMS	12
	Vehicle-Sham	15

**Table 2:** Percentage of mice in each estrus phase.

Phase	%
Proestrus	27.59
Estrus	26.44
Metestrus	12.64
Diestrus	33.33

**Table 3:** Mean (SD) plaque load by condition and major brain regions for 3× mice.

Genotype	Treatment	D Cortex	V Cortex	Septal Nuc	Subiculum	Hippoc	Hindbrain
ANA12	TMS	0.16 (0.28)	0.08 (0.16)	6.83 (6.47)	21.94 (19.51)	1.17 (1.85)	40.49 (42.35)
	Sham	0.31 (0.36)	0.31 (0.62)	9.28 (17.30)	32.67 (33.31)	0.43 (0.58)	15.15 (19.16)
Veh	TMS	1.21 (2.28)	0.16 (0.27)	18.18 (34.02)	22.02 (11.45)	0.62 (0.49)	12.92 (14.71)
	Sham	0.54 (0.80)	1.76 (3.37)	7.19 (8.52)	156.96 (176.38)	3.47 (4.53)	28.92 (24.30)

## Data Availability

The data that support the findings of this study can be found at: https://data.mendeley.com/datasets/kxmrcnwkpg/1 (accessed on 8 October 2024).

## Supplementary Materials

The following supporting information can be downloaded at: https://www.mdpi.com/article/10.3390/brainsci14121186/s1.
