# Supplementary material for: rTMS Modulation of Behavioral and Biological Measures in 3xTg-AD Mice
Source: Brain Sci. 2024 Nov 26;14(12):1186. doi: 10.3390/brainsci14121186 (PMC11674534; doi:10.3390/brainsci14121186)

Supplementary Table S1. Correlation coefficients between behavioral tasks and behavioral measurements. Pearson's coefficient was calculated for all measurements except for OFT % Center due to violation of normality, so Spearman's correlation was utilized. No coefficients were significant (all p's > 0.05). Pro = proestrust, Est = estrus, Met = metestrus, Di = diestrus, OFT = open field task, Dist = distance, ORT = object recognition task, Expl = exploration, OPT = open field task, Corr = correct.

Phase	OFT Dist	OFT % Cent	ORT Total Expl	OPT Total Expl	Y Entries	Y % Corr
Pro	-0.03	0.06	0.04	-0.16	-0.13	0.06
Est	0.00	-0.16	-0.06	0.03	0.06	0.05
Met	0.17	0.05	0.04	0.02	0.21	-0.03
Di	-0.14	-0.01	0.05	0.11	-0.14	0.01

Supplementary Figure S1. A) Unaltered Western blot image of TrkB and ß-actin. TrkB produces two bands (Full Length and Truncated) at 145 and 95 kDA. B) Unaltered Western blot image of AKT (40kDA)and Cofflin (18kDA). Images were acquired on the Odyssey Licor scanner. Images were acquired on the Odyssey Licor scanner.

A)

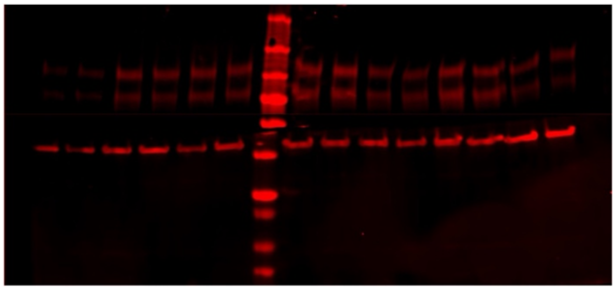

B)

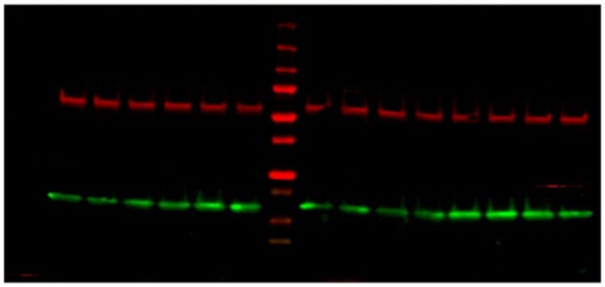

Supplement: Supplementary file 1 [file brainsci-14-01186-s001.zip › brainsci-3269482-supplementary.pdf]
